# Self-perceived versus objectively measured competence in performing clinical practical procedures by final year medical students

**DOI:** 10.5116/ijme.5709.2a7e

**Published:** 2016-04-28

**Authors:** Patricia Katowa-Mukwato, Sekelani Banda

**Affiliations:** 1 University of Zambia, School of Medicine, Department of Medical Education Development, Zambia

**Keywords:** Clinical competence, self-perceived, objectively measured competence, medical students, clinical practical procedures

## Abstract

**Objectives:**

To determine and compare the self-perceived and objectively measured competence in performing 14 core-clinical practical procedures by Final Year Medical Students of the University of Zambia.

**Methods:**

The study included 56 out of 60 graduating University of Zambia Medical Students of the 2012/2013 academic year. Self-perceived competence: students rated their competence on 14 core- clinical practical procedures using a self-administered questionnaire on a 5-point Likert scale. Objective competence: it was measured by Objective Structured Clinical Examination (OSCE) by faculty using predetermined rating scales. Rank order correlation test was performed for self-perceived and objectively measured competence.

**Results:**

Two thirds 36 (66.7%) of the participants perceived themselves as moderately competent, 15 (27.8%) rated themselves as highly competent while 3 (5.6%) had low self-perception. With objective competence, the majority 52 (92.8%) were barely competent while 4 (7.2%) were absolutely competent. When overall self-perception was compared to objectively measured competence, there was a discordance which was demonstrated by a negative correlation (Spearman rho -.123).

**Conclusions:**

Significant numbers of students reported low self-competence in performing procedures such as endotracheal intubation, gastric lavage and cardiopulmonary resuscitation which most never performed during the clinical years of medical education. In addition, the negative correlation between self-perceived and objectively measured competence demonstrated the inability of students to assess and rate themselves objectively due to fear that others may know their weaknesses and realize that they are not as competent as expected at a specific level of training.

## Introduction

Self-perception is the reported self-efficacy in performing a task.^1^ In the present  article, it is the reported ability to perform clinical practical skills, while objectively measured competence is the observed behaviour or practice.^2^ Competence of undergraduate medical students in performing basic clinical skills whether self-perceived or objectively measured has been investigated in Europe and North America with the former having been explored extensively.^3^^-^^8^ However there is little or no information about how students of African medical schools evaluate their core-competences.^7^ Similarly no such study had been conducted in Zambia prior to ours. This gap existed despite the evidence in literature that determination of the competency level of trainees at or near the point of graduation is critical as acquired competences are a central indicator of the quality of the curriculum.^7^ It was on the basis of this assertion that a study was conducted in 2013 to investigate the self-perception and objectively measured competency of final year medical students of the University of Zambia in performing 14 selected core-clinical and practical procedures.

The selected core-clinical and practical procedures were: IV cannula insertion, Nasogastric Tube (NGT) Insertion, gastric lavage, urethral catheterization, Cardiopulmonary Resuscitation (CPR), endotreacheal intubation, wound suturing, vaginal examination, examination of the placenta and lumbar puncture.  Other procedures were vaginal delivery, examination of the new-born, intramuscular drug administration and intravenous drug administration. The list was an amalgamation of essential procedures for undergraduate medical students by the United Kingdom General Medical Council^9^^,^^10^ and commonly encountered procedures at the Zambia’s University Teaching Hospital as identified by Banda, 2004.^11^

In assessing competence, previous studies have reported varying degrees of agreement between self-perceived and objectively measured competence in medical students.^8^^,^^12^^,^^13^ Colberly and Goldenhar in a cross-sectional survey aimed at assessing Acting Interns’ (Fourth year medical students at University of Cincinnati- United States of America) experience with and perceived level of competence performing six basic medical procedures, established that a vast majority of Fourth-Year Students reported not performing four (phlebotomy, intravenous catheter insertion, lumbar puncture and Foley catheter insertion).^3^ The six basic procedures were phlebotomy, intravenous catheter insertion, Arterial Blood Gas (ABG), nasogastric tube insertion, Foley catheter insertion and Lumbar Puncture. Procedure performance ranged from nine percent for Foley catheter to 50% for Arterial Blood Gasses (ABG).

The most frequently performed procedures in the Colberly and Goldenhar, study, were ABG and nasogastric tube insertion. Feeling of competency varied from 12% for Lumbar Puncture to 82% for Foley catheter insertion.^3^ Apart from Lumbar Puncture, majority of students felt competent performing other stated procedures without supervision by the end of the second acting intern rotation (ranging from 53% for intravenous insertion to 82% for Foley’s catheter insertion).^3^

Studies conducted on third year medical students and residents have revealed that self-assessment of competence correlates with frequency of procedure performance.^14^^,^^15^ For example Hicks and collegues,^15^ revealed that internal medicine residents reported needing 6-10 LP experiences to reach a “comfortable threshold” defined as the number of procedures at which two thirds of the house staff reported being comfortable or very comfortable performing. There is however some opposing views to this stance. For example Lai, Sivalingam and Ramesh in a study of medical students’ progress in self-perceived clinical competence, and relationship between experience and confidence in practical skills indicated that the correlation between experience and self-perceived competence was at best moderate.^4^

Based on the moderate correlation, Lai and his colleagues suggested that an increase in the student’s experience with a skill might not be accompanied by the same degree of increase in their self-perceived competence.  Factors that have been suggested to facilitate development of self-confidence include direct supervision and feedback, deliberate practice and having a dedicated procedure course to teach clinical skills.^4^^,^^16^^-^^19^

Self-perception of competence among medical students has also been assessed across different cultures. Barbosa and colleagues evaluated the self-perceived competence of medical students in three different countries: Portugal and two African Portuguese speaking countries Angola and Mozambique.^7^ The evaluation was across six domains of personal attitude, professional behavior, knowledge, clinical skills, general skills, and communication skills. The domain with the highest score across countries was personal attitude and professional behavior. Apart from Mozambique, clinical skills in Angola and Portugal received the lowest scores. The investigator assumed that the low values assigned to acquisition of competence in clinical skills could be due to students’ fear of making mistakes or the limited opportunities for practice during training.  

Barbosa and colleagues’ study like others cited above focused on self-reported competence, which is neither objective nor free from bias. Despite knowing what a student thinks about what she/he is capable of doing; self-perception is different from real or observed performance. Literature indicates poorer correlation between self-perceived and objectively measured competence in practical skills and comparatively better correlation in the “soft” skills like communication.^3^^,^^8^^,^^13^^,^^20^ As Eva and Regehr,^21^ suggested; the fundamental cognitive limitation in the ability of humans to know themselves as others see them restricts the usefulness of results of self-assessment.

Despite the subjectivity associated with self-perception in determining performance, Eva and Regehr, on the other hand asserted that self-assessment has two important functions both as a mechanism for identifying one’s weaknesses and as a mechanism for identifying one’s strengths.^21^ Each of these mechanisms having distinct and complementary functions. As a mechanism for identifying weaknesses or gaps in one’s skills and abilities, self-assessment serves several potential functions. Firstly, in daily practice, the identification of one’s weaknesses allows the professional to self-limit in areas of limited competence. For example, in some circumstances the professional can quickly reject certain assignments because they are able to recognize that they are unlikely to complete the tasks. In other circumstances, an individual can realize that the task is beyond his ability, and can decide to consult or refer the problem to another individual. The ability to recognize tasks beyond one’s competence is critical especially in the medical field where trial and error has no room as practitioners deal with people’s lives.

In addition positive self-efficacy has been related to persistence, tenacity, and achievement in educational settings.^1^ Therefore it can be contended that medical students with high self-perception towards a task are more likely to devote their energies towards learning such a task, consequently increasing the numbers of attempts with increased likelihood of success with each additional attempt leading to increased confidence, and improved performance.

Another dimension of self-perception of competence that has been reported in literature is that of comparisons between self-perceptions of medical students and that of their teachers. Sicaja, Romic and Prka evaluated self-assessed level of competence of graduating medical students at Zagreb University School of Medicine in 99 clinical skills, and compared it with the level expected by their teachers and those defined by a criterion standard.^22^ The findings were that students’ perception of their own achievement differed from their teachers’ expectations. Students tended to assess their skills much lower than expected by their teachers and published criterion standard. Similar findings have been reported from Great Britain, Denmark, Netherlands, Belgium and the United States.^23^^-^^25^ Similar to other studies, factors that were associated with higher scores in Sicaja, Romic and Prka study were better organization of clinical skills teaching in those subjects, having taken an additional clinical skills course and student interest.^22^

Compared to self-perceived competence, objectively measured competence among medical students has not been extensively assessed. Sacaja, Romic and Prka cited the cost of tools that can measure competence such as OSCE as a hindrance.^22^ However Elango and colleagues objectively measured the clinical skills of undergraduate students in Malaysia.^26^ In this Malaysian study, a list of practical skills that students should be competent in were identified. The skills were demonstrated to students by academic staff/nurses using manikin in clinical skills unit. Students were then given opportunity to practice under supervision. Later they carried out the skills on actual patients in wards still under supervision. During the end of semester examination at 4th and 5th year level, students were assessed on the list of skills as part of Objective Structured Practical Examination (OSPE) using a structured checklist.

The major finding was that failure rate in practical skills was high in most of the station (seven out of eight). It is however interesting to note that despite of formal training in basic practical skills, many students failed. The investigators concluded that assessment of practical skills as part of composite examination does not ensure that all students acquire adequate level of competence, because clinical skills training require time and practice and that training should be monitored and skills be assessed through dedicated formative assessments.

Considering the documented weaknesses and subjectivity of self-reports that emanates from the fundamental cognitive limitation in the ability of humans to know themselves as others may see them,^21^ and the fact that medical students may overestimate or underestimate their clinical performance,^27^ our study investigated the following question “How does self-perception of competence compare with objectively measured competence in selected clinical practical procedures among University of Zambia Final Year Medical Students in the last six months of training?” Our study therefore measured both the self-perceived (self-reported) and the objectively measured competency, as a means for the later to off-set the weaknesses of the former.

## Methods

### Study design

The study utilized a cross sectional survey design involving a class of 60 graduating University of Zambia Medical Students of the 2012/2013 academic year.

### Data collection

#### Self-perception of competence

Fifty-six (56) students from a class of 60 (93% response rate) of the final year medical students’ of 2012/2013 completed a questionnaire that required them to rate their self-perception of competence on a 5-point Likert scale rating on 14 clinical procedures : 1) Intravenous cannula insertion, 2) naso-gastric tube insertion, 3) gastric lavage, 4) urethral catheterisation, 5) lumbar puncture, 6) cardiopulmonary resuscitation, 7) endotracheal intubation, 8) wound suturing, 9) vaginal examination, 10) normal vaginal delivery, 11) examination of the placenta, 12) examination of the new born, 13) intravenous drug administration, and 14) intramascular drug administration. For each procedure, a student selected a corresponding level of perceived competence in performing it from a five point Likert scale (Lai, Sivalingam, and Ramesh, 2007). The Scale ranged from; (1) grossly inadequate, (2) knowing approach only in theory, (3) not confident in real situation, only competent in making certain decisions, needs seniors on constant standby, (4) reasonably competent, but needs seniors who are contactable for consultation, and (5) very competent, can be relied on without supervision. The five levels of self-perception were later reduced to three for the purposes of data analysis; low self-perception for level one and two, Moderate self-perception for level three and four and high self-perception for level five.

In addition, for each of the procedures included in the self-perception of competence questionnaire, students concurrently rated the frequency of experience on a 5-item Likert scale; (1) never performed, (2) performed 1-5 times, (3) for  6-10 times, (4) for 11-20 and (5) for more than 20 times. The five point Likert scale of experience was similarly reduce to three categories for the purposes of data analysis. With regard to the 14 selected skills, it is expected that in the last six months of undergraduate medical training, all students should be at least on level 3 for number of attempts at a particular procedure and level 4 of self-perceived competence on all items.

### Objectively measured competence

It was measured in the final examination OSCE held at the end of the 2012/2013 academic year for the final year medical students. Fifty-six (56) of the 60 students (93% response rate) consented to participate in our study. In the final OSCE, there were only seven practical stations.

Therefore, from the 14 procedures that were utilised in estimation of self-perceived competence, the investigator successfully negotiated to have three of the procedures included among the seven practical stations. The investigator was aware of the need not to alter significantly by way of interfering in the natural settings of the OSCE. Since OSCEs are the prescribed means by which competency levels of students are assessed, the measure of objective competence in our study therefore needed to be as close to reality as possible rather than stage managed.

The three procedures utilized in the study were, CPR, insertion of a NGT and intravenous drug administration. On all the practical OSCE stations, students were required to “show how” a procedure is done using a manikin. In addition to showing how, students were also require to give a running commentary of the requirements during the performance of the procedure, structures involved and the standard practice. Being a final examination, clinical experts in the clinical departments determined the OSCE stations, observed and scored the performance of students using structured checklist as normally done during OSCEs. The pass mark for OSCE was determined by the School at 50%.

**Table 1:** Levels of self-perceived competence on 14 selected practical procedures n=56

Procedure	Level of self-perceived competence			No Response n (%)
	Low self-perception n (%)	Moderate self-perception n (%)	High self-perception n (%)	
Intravenous cannula insertion	0 (0.0)	23 (41.1)	31 (55.4)	2 (3.6)
Normal vaginal delivery	0 (0.0)	26 (46.4)	28 (50)	2 (3.6)
Vaginal examination	0 (0.0)	31 (55.4)	21 (37.5)	4 (7.1)
Urethral catheterization	0 (0.0)	31 (55.4)	23 (41.1)	2 (3.6)
Intramuscular drug administration	2 (3.6)	27 (48.2)	21 (37.5)	6 (10.7)
Intravenous drug administration	4 (7.1)	29 (51.8)	23 (41.1)	0
Examination of the placenta	4 (7.1)	20 (35.7)	26 (46.4)	6 (10.7)
Examination of the new-born	9 (16)	27 (48.2)	20 (35.7)	0
Lumbar puncture	8 (14.3)	39 (69.6)	5 (8.9)	4 (7.1)
Nasal Gastric tube insertion	18 (32.1)	33 (58.9)	5 (8.9)	0
Wound suturing	11 (19.6)	34 (60.7)	8 (14.3)	3 (5.4)
Endotreacheal intubation	28 (50)	23 (41.1)	1 (1.8)	4 (7.1)
Gastric Lavage	33 (58.9)	14 (25)	2 (3.6)	7 (12.5)
Cardiopulmonary Resuscitation	34 (60.7)	20 (35.7)	2 (3.6)	0

### Data analysis

#### Self-perceived competency

Responses on self-perception of competence were entered into SPSS version 17. Frequencies for the three levels of self-perception (low, moderate and high self-perception) were computed. Findings on overall self-perceived competence on individual procedure are presented in Table 2. Rank order correlations (Spearman rho) test was performed to test for association between self-perception of competence and objectively measured competence.

**Table 2:** Cross tabulation and correlation between overall objective competence (OSCE) performance levels and overall self-perceived competence

Objective -competence (OSCE Performance)	Self-perceived competence			Total	*p *value
	Low self-perception n (%)	Moderate self-perception n (%)	High self-perception n (%)		
Not competent	0 (0)	0 (0)	0 (0)	0 (0)	.451 rho -.123.
Barely competent	3 (100)	32 (88.9)	15 (100)	50 (92.6)	
Absolutely competent	0(0)	4 (11.10	0 (0)	4 (7.4)	
Total	3 (100)	36 (100)	100 (0)	54 (100)	

#### Objectively measured Competency

Results of OSCE for each candidate were entered onto SPSS as percentages scores (internal level) and frequencies for pass/fail rate were computed using the school pass mark of 50%. To facilitate rank order correlation between self-perceived and objectively measured competence, OSCE scores which was originally at interval level in percentage form were re-categorized from the specific numeric scores in percentage form to ordinal level with three categories. The categories were; not competent (Score of 0-49%), barely competent (Score of 50-75%) and absolutely competent (Score 76 to 100%). Following re-categorization, frequencies for levels of objective competence (not-competent, barely-competent and absolutely- competent) on three practical procedures included in OSCE were also computed. Re-categorization of OSCE scores meant that the two variables; objective and self-perceived competence were now all at ordinal level of measurement. Consequently, rank order correlations (Spearman rho) test was performed between self-perceived and objective competence Table 2.

## Results

### Self-perception of competence

Overall, two thirds 36 (66.7%) of the participants perceived themselves as moderately competent in performing the 14 selected practical procedures, 15 (27.8%) rated themselves as highly competent while only 3 (5.6%)  had low self-perception.  With regard to the individual procedural skills (Table 1), none of the participants had low self-perception of competence on four out of the 14 selected practical procedures, instead they had either moderate or high self-perception. High self-perception was reported on insertion of intravenous cannula and in conducting normal vaginal deliveries with at least 50% of students perceiving themselves as highly competent.

**Table 3:** Cross tabulation and correlation between objective competence (OSCE) performance levels and self-perceived competence for the three practical procedures*

Practical procedures	Manifest - competence (OSCE performance)	Self-perceived competence			Total	p value and Spearman rho
		Low self-perception n (%)	Moderate self-perception n (%)	High self-perception n (%)		
Cardiopulmonary resuscitation	Not competent	13 (38.2)	9 (45)	2 (100)	24 (42.9)	p value 0.270 rho -.150
	Barely competent	10 (29.4)	6 (30)	0 (0)	16 (28.6)	
	Absolutely competent	11 (32.4)	5 (25)	0 (0)	16 (28.6)	
	Total	33 (100)	2 (100)	2 (100)	56 (100)	
Nasogastric tube insertion	Not competent	0 (0)	7 (21.2)	0 (0)	7 (12.5)	p value 0.346 rho .128
	Barely competent	9 (50)	7 (21.2)	0 (0)	16 (28.6)	
	Absolutely competent	9 (50)	19 (57.6)	5 (100)	33 (58.9)	
	Total	18 (100)	33 (100)	5 (100)	56 (100)	
Intravenous drug administration	Not competent	0 (0)	2 (6.9	3 (13)	5 (8.9)	p value 0.000 rho -.521
	Barely competent	3 (75)	7 (24.1)	20 (86)	30 (53.6)	
	Absolutely competent	1 (25)	20 (69)	0 (0)	21 (37.5)	
	Total	4 (100	29 (100)	23 (100)	56 (100)	

*Since students’ identification numbers were not utilized, all computed correlations were done at group and not individual level.

The lowest self-perception was in performing CPR where 34 (60.7%) of participants had low self-perception, followed by gastric lavage at 33 (58.9%) and endotracheal intubation at 28 (50%).  Moderate self-perception of competence was recorded for lumbar puncture (n-39, 69.6%), wound suturing (n=34, 60.7%), nasogastric tube insertion (n=33, 58.9%), vaginal examination and urethral catheterisation (n=31, 55.4% each). These are common day-to-day clinical practical procedures performed on the ward and it is therefore worrisome that mastery, measured with self-perception, was not universal.

### Number of times procedures were performed

With self-rated Experience, it was gratifying to note that all students had performed common procedures such as intravenous cannula insertion and normal vaginal deliveries at least 6 times during the clinical years with more than 80% performing the two procedures more than 10 times. To the contrary a significant proportion of the final year medical students had never performed common and life-saving procedures such as CPR (n=27, 48.2%), endotracheal intubation (n=30, 53.6%), and gastric lavage (n=36, 64.3%). 

### Objectively measured competence

There were varying levels of objective competence in performing the three practical procedures that were included among the seven OSCE stations. CPR, recorded a highest number of students who were not competent (n=24, 42.9%).  More than half (n=30, 53%) were barely competent in intravenous drug administration while majority 33 (58.9%) were absolutely competent in insertion of NGT. It is worrying to note that at the end of the undergraduate medical training, a good proportion of students 24 (42.9%), 7 (12.5) and 5 (8.9) were still not competent in the three commonly encountered procedures of CPR, NGT insertion and IV drug administration. Apart from the three procedures being required for day-to- day practice of junior doctors, two of the three CPR and IV drug administration are lifesaving skills necessary for patient survival.

Further analysis of self-perceived and objectively measured competence of the three clinical practical procedures which were included in the OSCE revealed negative correlation between self-perceived and objective competence for two out of the three procedures Table 3. The correlations (Spearman rho) between self-perceived and objective competence were as follows: Cardiopulmonary Resuscitation (-.150, p value 0.270); Intravenous Drug Administration (-.521, p value 0.000) and Nasogastric Tube Insertion (.128, p value 0.346). Although the correlation (rho=0.128) between objective and self-perceived competence was positive for NG Tube insertion, it was rather weak.

In order to testing for overall association between objective and self-perceived competency, a correlation test between the two variables was performed as shown in Table 2. Upon computation, there was a negative correlation between self-perceived and objective competence (OSCE performance). Spearman rho -.123 and p value .451. All the 15 (100%) participants who rated themselves as highly competent under self-perception of competence were categorized as barely competent with regard to performance in practical procedures during their OSCE.

## Discussion

### Self-perceived competence

In this article, self-perception of competence is operationally defined as the reported ability to perform clinical practical skills. Self-perception of competence was assessed for individual practical procedures and as aggregate for all 14 selected practical procedures. On individual procedures, significant numbers of students reported low-self-perception in performing three out of the 14 selected procedures: CPR, gastric lavage and endotracheal intubation (Table 1). The finding of low self- perception of competence on certain procedural skills is not unique to our project as similar observations have been made by other researchers before. For example Barbosa and colleagues in a study to evaluate the self-perceived competence of medical students in three different countries; Portugal, Angola and Mozambique, they reported that apart from Mozambique, clinical skills in Angola and Portugal received the lowest scores.^7^ The investigators assumed that the low values assigned to acquisition of competence in clinical skills could be due to the limited opportunities for practice during training.  This signifies the importance of dedicated clinical skills training throughout the duration of medical education.  The role of dedicated clinical courses have been highlighted by other researchers such as Promes and colleagues who reported that first year residents who completed a dedicated procedure course in medical school were significantly more likely to report adequacy in performing basic medical procedures.^6^

A positive finding from our study was that all the students had either moderate or high self-perception on intravenous cannula insertion, normal vaginal deliveries, vaginal examination and urethral catheterization (Table 1).  Apparently the four procedures (intravenous cannula insertion, normal vaginal deliveries, vaginal examination and urethral catheterization) in consecutive order were performed more times with all students reporting performing IV cannula insertion and normal vaginal deliveries more than 6 times. The high numbers of times these procedures were performed could have contributed to the improved levels of self-perception related to the same procedures. Previous studies have reported associations between number of time a procedure was performed and self-assessed competence.^6^

The motivation for students to conduct more vaginal deliveries could be due to the fact that patients for the procedure are readily available considering that the class of students investigated in our study learnt all the procedures at the bed side. Similarly most students could have inserted more IV cannula due to the readily availability of patients indicated for the procedure. In a setting were clinical practical procedures are sorely learnt from the bedside on actual patients, whether a students perform or does not perform a procedure is to some extent dependent upon availability of patients requiring such a procedure. This fact has been highlighted by other researchers for example Neilsen and colleagues who stated that 99% of students had to work hard and be active in order to get access to practice skills in a clinical setting, but only, 36% had the chance to practice to the extent they wanted.^33^

Other procedures on which self-perception of competence were assessed included; intramuscular drug administration, intravenous drug administration, Lumbar Puncture, nasogastric tube insertion and wound suturing (Table 1). Studies in the past including one conducted by Colberly and Goldenhar, have reported low levels of self-perception on LP, with only 25% reporting feeling competent performing it without supervision.^3^ This was not the case for our study, as majority reported feeling moderate and very competent respectively compared to only 14.3% who reported low self-perception in performing LP (Table 1).

### Objectively measured competence

Actual performance also referred to as “shows how” in our study, was assessed using results of the final year Objective Structured Clinical Examination (OSCE). It should be noted that there were variations in the procedures included in the OSCE and self-perception questionnaire. As opposed to the 14 selected clinical practical procedures included in the self-perception of competence questionnaire, only seven practical procedures were included in the final OSCE. From the seven practical OSCE stations, the investigator successfully negotiated to have three of the procedures included in the OSCE. Given that only seven stations were dedicated to practical procedures, it was acceptable to investigate three out of seven. Additionally the investigator was conscious not to interfere with the setting of the final examination. To compensate for the variation in procedures included in the OSCE and self-perception questionnaire, the three procedures included in the OSCE underwent detailed result analysis with regard to knowledge of students, self-perception of competency, manifest-competency and frequency of practice.

The three practical OCSE station required students to “show how” a procedure is done using a manikin. In addition, students were also required to give a running commentary of the requirements during the performance of the procedure, structures involved and the standard practice. Being a final examination, clinical experts in the departments determined the procedures, observed and scored the performance (manifest-competence) of students using structured checklist as normally done during OSCEs. When results were aggregated, all the students passed the OSCE. One plausible reason for the high overall pass rate in the OSCE was the presumably “low” school pass mark of 50%.  Raising the pass mark would have clearly reduced the pass rates. Although all students passed the OSCE (using criterion referencing), majority were simply barely competent.

When the three practical procedures included in the OSCE were compared, there were varying levels of objective competence in performing them. Among the three procedures, CPR recorded a highest number of students who were not competent followed by NGT insertion and intravenous drug administration. It is worrying to note that at the end of the undergraduate medical training, a good proportion of students were still not competent in the three commonly encountered procedures of CPR, NGT insertion and IV drug administration. Apart from the three procedures being required for day-to- day practice of junior doctors, two of the three CPR and IV drug administration are lifesaving skills required during emergencies, and therefore necessary for patient survival.  As stated earlier, the fact that the two procedures are required during emergencies, where trial and error by students is not acceptable.^34^^,^^35^ and typically for CPR, almost half of the  students never performed it during the three years of clinical medical education consequently the observed low level of objective competence.

When the three practical procedures included in the OSCE were correlated in terms of objective and self-perceived competence, there was negative correlation on two out of the three (Table 3). Similarly, there was also a negative correlation between objectively measured competence (all practical procedures in the OSCE) and self-perceived competence (all practical procedure in the self-perception questionnaire) Table 2.

Similar to findings of our study, previous studies have reported varying degrees of agreement between self-perceived and objectively measured competence in medical students.^5^^,^^13^^,^^20^^,^^21^ As indicated, in our study, there was lack of associations between, self-perceived competence and objective competence. All the students who perceived themselves as highly competent were categorized as barely competent with regard to performance in practical procedures during their OSCE.

Several reasons could be advance for these disagreements. Firstly self-reports are more subjective, and therefore it is generally accepted that competency may be better assessed using Objective Structure Clinical Examination (OSCE). The subjectivity of self-reports as an assessment tool is also supported by Eva and Regehr in their assertion that the fundamental cognitive limitation in the ability of humans to know themselves as others see them restricts the usefulness of self-assessment results.^21^ Secondly, medical students have been shown to both overestimate and underestimate their clinical performance.^27^ Therefore the reported high self-perception could not be a true reflection.  Although it can be argued that not all procedures in the self-perception of competence questionnaire were in the OSCE, on the contrary, when correlations were performed between self-perceived and objective competence for the three practical procedures which were in both the, negative correlations were also recorded for two out of the three.

Our study was one of the few unique ones that studied and compared objective and self-perceived competence in clinical practical procedures. Medical education literature alerts us that in comparison to self-perceived competence, objective competence among medical students has not been extensively investigated. Sicaja, Romic and Prka cited the cost of tools that can measure competence such as OSCE as a hindrance.^22^ Comparable to our study, Elango, and colleagues objectively measured the clinical skills of undergraduate students in Malaysia.^26^ As opposed to the high OSCE pass rates in our study, the major finding in the Malaysian study was that failure rate in practical skills was high in most of the station (7 out of 8).  This finding was despite the skills having been demonstrated to students and an opportunity to practice on manikins and later on patients under supervision accorded. It’s however worthy noting that the pass mark for the Malaysian study is not provided thus accurate comparisons with regard to pass rates between the our study and the Malaysian one could not be made.

### Limitations of the reported findings

The most significant limitation of the reported findings is that the clinical procedures in the self-perception and the objective competence assessment could not identically matched because the objective  competence was measured in final OSCE in which it was undesirable to interfere with the content and number of practical stations. However, we believe the findings provide enough credibility and fidelity because the three practical procedures from the self-perception questionnaire which were assessed in the OSCE (objective competence) underwent direct comparisons in term of self-perception and objective competence.

## Conclusions

Two thirds of the participants perceived themselves as moderately competent in performing the 14 selected practical procedures. On individual procedures, significant numbers of students reported low self-competence in performing procedures such as endotracheal intubation, gastric lavage and CPR which most never performed during the three years of clinical medical education despite the procedures being taught. Regarding objectively competence, despite all students passing the Objective Clinical Practical Examination (OSCE) using the school pass mark of 50% the majority of were barely competent.  The study revealed a negative correlation (discordance) between overall objective and self-perceived competence. The discordance between self-perceived and objective competence implies the need for medical educators to be aware of the inability of students to assess and rate themselves objectively due to fear that others may know their weaknesses and realize that they are not as competent as expected at a specific level of training. This therefore confirms the limitation in the use of self-assessment results and supports the use of objectively measured competency.

### Acknowledgements

I acknowledge the University of Zambia School of Medicine for the opportunity to pursue my doctoral studies and The National Institutes of Health (NIH) through the Medical Education Partnership Initiative (MEPI) programmatic award No. 1R24TW008873 entitled Expanding innovative multidisciplinary medical education in Zambia for the financial support towards the project.

### Conflict of Interest

The authors declare that they have no conflict of interest.
